# Metabolic Reprogramming of Breast Tumor-Educated Macrophages Revealed by NMR Metabolomics

**DOI:** 10.3390/cancers15041211

**Published:** 2023-02-14

**Authors:** Ana S. Dias, Catarina R. Almeida, Luisa A. Helguero, Iola F. Duarte

**Affiliations:** 1 CICECO—Aveiro Institute of Materials, Department of Chemistry, University of Aveiro, 3810-193 Aveiro, Portugal; 2 iBiMED—Institute of Biomedicine, Department of Medical Sciences, University of Aveiro, 3810-193 Aveiro, Portugal

**Keywords:** breast cancer, tumor microenvironment (TME), tumor-associated macrophages (TAM), cell metabolism, metabolic reprogramming, NMR metabolomics

## Abstract

**Simple Summary:**

Tumor-associated macrophages (TAM), which constitute the most abundant immune cells in the breast tumor microenvironment (TME), display immune-suppressive functions that promote breast cancer (BC) progression and are associated with poor disease outcomes. Altered metabolism is recognized both as a hallmark of tumor cells and an important determinant of macrophage functional regulation. Hence, characterizing the metabolic crosstalk between tumor cells and TAM represents an attractive approach to discovering new ways of modulating the complex TME towards favorable anti-tumor immunity. The present study elucidates how in vitro generated tumor-educated macrophages (TEM) reprogram their metabolism and phenotype in response to the metabolic conditions imposed by different BC cell subtypes. Several metabolites and metabolic pathways with potential immunoregulatory roles are highlighted, expanding current knowledge on the metabolic–phenotypic axis in TEM.

**Abstract:**

The metabolic crosstalk between tumor cells and tumor-associated macrophages (TAMs) has emerged as a critical contributor to tumor development and progression. In breast cancer (BC), the abundance of immune-suppressive TAMs positively correlates with poor prognosis. However, little is known about how TAMs reprogram their metabolism in the BC microenvironment. In this work, we have assessed the metabolic and phenotypic impact of incubating THP-1-derived macrophages in conditioned media (CM) from two BC cell lines cultured in normoxia/hypoxia: MDA-MB-231 cells (highly metastatic, triple-negative BC), and MCF-7 cells (less aggressive, luminal BC). The resulting tumor-educated macrophages (TEM) displayed prominent differences in their metabolic activity and composition, compared to control cells (M0), as assessed by exo- and endometabolomics. In particular, TEM turned to the utilization of extracellular pyruvate, alanine, and branched chain keto acids (BCKA), while exhibiting alterations in metabolites associated with several intracellular pathways, including polyamines catabolism (MDA-TEM), collagen degradation (mainly MCF-TEM), adenosine accumulation (mainly MDA-TEM) and lipid metabolism. Interestingly, following a second-stage incubation in fresh RPMI medium, TEM still displayed several metabolic differences compared to M0, indicating persistent reprogramming. Overall, this work provided new insights into the metabolic plasticity of TEM, revealing potentially important nutritional exchanges and immunoregulatory metabolites in the BC TME.

## 1. Introduction

In many solid tumors, macrophages represent the most abundant cell population of the immune infiltrate. Depending on the microenvironmental conditions, which change during tumor progression and differ between tumor regions, tumor-associated macrophages (TAMs) can display anti-tumoral activity, associated with a pro-inflammatory (M1-like) phenotype, or support tumor growth and invasiveness by acquiring an immuno-suppressive phenotype that is closer to anti-inflammatory (M2-like) macrophages [1]. The dominance of M2-like pro-tumorigenic TAMs in the tumor microenvironment (TME) has been correlated with poor prognosis and drug resistance in a variety of human malignancies [2,3,4,5]. In patients with BC, tumor-associated macrophages (TAMs) can account for up to 50% of the tumor weight [6], with a high intra-tumoral density of M2-like macrophages (typically CD163^+^) being positively correlated to larger tumor size, unfavorable prognostic factors (e.g., higher grade, proliferating index, hormone receptor negativity) and reduced survival [7,8,9,10].

Several microenvironmental factors in the tumor niche, including chemokines, cytokines, and growth factors, are recognized to influence macrophage plasticity and promote their pro-tumorigenic programming [11]. Recently, changes in metabolic pathways and associated metabolites in the TME have also emerged as possible key determinants of the phenotype and functional behavior of TAMs [12]. Under the regulation of oncogenes and tumor suppressors, tumor cells typically display a profoundly altered metabolism to support their enhanced energetic and biosynthetic needs [13]. Simultaneously, tumor cells engage in a metabolic interplay with the surrounding stromal cells, thereby shaping their metabolism and functions [14,15]. Indeed, tumor-derived metabolites may dictate the pro-tumoral differentiation of TAMs, as exemplified by lactate [16,17,18], retinoic acid [19], and β-glucosylceramide [20]. On the other hand, TAM-secreted metabolites can also be important in the context of tumor progression and response to treatment. This is clearly illustrated by the work of Halbrook et al., where pyrimidine nucleosides released by macrophages cultured in a medium from pancreatic ductal carcinoma (PDA) cells were found to confer gemcitabine resistance to PDA cells, via competitive inhibition at deoxycytidine kinase [21]. Hence, characterizing the metabolic crosstalk between tumor cells and TAMs represents an attractive approach to discovering new ways of modulating the complex TME towards favorable anti-tumor immunity.

In this work, we aimed to assess how macrophages change their metabolism and phenotype in response to the metabolic microenvironment created by BC cells. For that purpose, the media resulting from previous culture of MDA-MB-231 cells, widely taken as a model for highly metastatic, triple-negative BC, and of estrogen-responsive MCF-7 cells, representative of the BC luminal subtype, were used to condition human macrophages derived from the THP-1 cell line. By being exposed to the nutrients and secreted factors that result from BC cells activity, the resulting macrophages represent an in vitro model of the TAMs found in the BC TME and are hereby designated as tumor-educated macrophages (TEMs). For each BC cell type, given the well-established role of hypoxia in modulating BC metabolism and pathological features [22,23], conditioned media (CM) were collected after incubation in both normoxic and hypoxic conditions. Then, we employed untargeted NMR profiling of the culture media (exometabolomics) and of cell extracts (endometabolomics) to identify possible nutritional exchanges between BC and TEMs and to reveal the TEMs’ metabolic adaptations. The results highlight several metabolites with potential immuno-modulatory roles and provide novel clues on the metabolic plasticity of TEMs, as well as on how this can be influenced by the specific metabolic programs of different BC subtypes.

## 2. Materials and Methods

### 2.1. Cell Culture

The human BC cell lines MDA-MB-231 and MCF-7 and the THP-1 human monocytic cell line were obtained from the American Type Culture Collection (ATCC). BC cells were grown in Roswell Park Memorial Institute (RPMI-1640) medium (Thermo Fisher Scientific, Bremen, Germany) supplemented with 10% fetal bovine serum (FBS, Thermo Fisher, Waltham, MA, USA) and antibiotics (50 U/mL penicillin and 50 µg/mL streptomycin). The monocytic cell line was cultured in the same medium, additionally supplemented with 1 mM sodium pyruvate (Gibco, Grand Island, NY, USA). To obtain macrophages, THP-1 monocytes were incubated for 24 h with 50 ng/mL phorbol 12-myristate 13-acetate (PMA, Merck-Sigma Aldrich, St. Louis, MO, USA), followed by a 24 h resting period in fresh RPMI-1640 medium containing 10% FBS. All cell cultures were tested for mycoplasma infection by PCR and were maintained at 37 °C in a 5% CO_2_ atmosphere.

### 2.2. Collection of Breast Cancer (BC) Cells-Conditioned Medium (CM)

**Figure 1:**
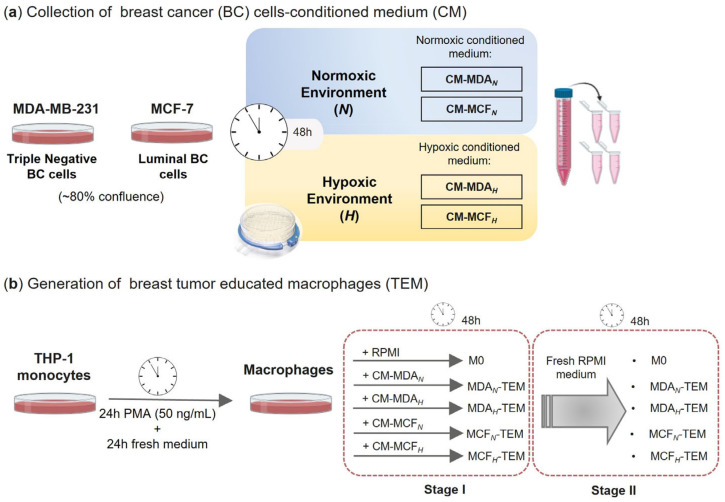
Schematic overview of the experimental design: (**a**) collection of conditioned media (CM) from MDA-MB-231 or MCF-7 breast cancer (BC) cells, incubated for 48 h, under normoxia (21% O_2_) or hypoxia (5% O_2_); (**b**) in vitro generation of breast tumor-educated macrophages (TEM) through 48 h incubation of THP-1-derived macrophages in BC cells-CM (Stage I), followed by re-incubation in fresh RPMI medium (Stage II). PMA, phorbol 12-myristate 13-acetate; RPMI, Roswell Park Memorial Institute medium (with 10% FBS).

Breast cancer cells at 80% confluence were cultured in RPMI-1640 medium containing 10% FBS and kept in a normoxic (*N*, 21% O_2_) or hypoxic (*H*, 5% CO_2_, and 95% N_2_) atmosphere, the latter being provided in a hypoxia incubator chamber (STEMCELL Technologies, Vancouver, BC, Canada). After 48 h incubation, conditioned media (CM) were collected, centrifuged for 5 min at 1000× *g* to remove cell debris, aliquoted, and stored at −80 °C (Figure 1a). CM retrieved from MDA-MB-231 and MCF-7 cell cultures are identified as CM-MDA*_N/H_* and CM-MCF*_N/H_*, respectively. For macrophage incubations, a pool with CM aliquots from at least three cell passages grown independently was prepared and sterilized using a 0.2 μm filter.

### 2.3. In Vitro Generation of Tumor-Educated Macrophages (TEMs)

As schematically depicted in Figure 1b, tumor-educated macrophages (TEMs) were generated by incubating THP-1-derived macrophages for 48 h (Stage I), in normoxic conditions, at a density of 1 × 10^6^ cells/mL in: (i) RPMI-1640 medium containing 10% FBS (control macrophages, M0, that represent the initial state of monocyte-derived macrophages, before activation by the TME signals); (ii) CM from normoxic MDA-MB-231 cells (MDA*_N_*-TEM); (iii) CM from hypoxic MDA-MB-231 cells (MDA*_H_*-TEM); (iv) CM from normoxic MCF-7 cells (MCF*_N_*-TEM); (v) CM from hypoxic MCF-7 cells (MCF*_H_*-TEM). Moreover, to assess the persistence of CM-induced changes, a second stage was considered (Stage II), where cells were transferred into fresh unconditioned RPMI-1640 medium (+10% FBS) and incubated for an additional 48 h period.

### 2.4. Samples Collection and Preparation for NMR Metabolomics

THP-1-derived macrophages were cultured in 100 mm diameter Petri dishes in the conditions described above. Three independent assays, each with two replicates, were performed, giving a total of six samples per condition (except for incubations with hypoxic media, where four replicates were retrieved). At the end of Stage I and Stage II (defined above), the medium was collected and immediately frozen, while each dish was washed four times with ice-cold PBS and the cells extracted, according to a procedure previously described [24]. Briefly, 800 µL of cold methanol 80% were added, the cells were scrapped off into microtubes containing 0.5 mm glass beads and the suspension vortexed for 2 min. This was followed by addition of chloroform (640 µL) and ultrapure water (288 µL), intercalated by 2 min vortexing, and final centrifugation (10,000× *g*, 15 min, 4 °C). The polar and non-polar phases obtained were then separated and dried in a Speedvac concentrator 5301 (Eppendorf, Hamburg, Germany) or under a nitrogen flow, respectively. All extracts were stored at −80 °C until NMR analysis. As for the medium samples, after thawing and centrifugation (1000× *g*, 5 min), 300 µL of supernatant was mixed with 600 µL cold methanol, and the mixture was kept at −20 °C for 30 min to precipitate proteins. Medium supernatants were then recovered by centrifugation (13,000× *g*, 20 min), dried under vacuum, and stored at −80 °C. Acellular media, incubated in parallel under the same conditions, were also processed as described above. For NMR analysis, dried medium and polar extracts were reconstituted in 600 µL of deuterated phosphate buffer (PBS 100 mM, pH 7.4) containing 0.1 mM 3-(trimethylsilyl) propionic acid (TSP-d_4_), whereas non-polar extracts were dissolved in 600 µL of deuterated chloroform containing 0.03% tetramethylsilane (TMS). Samples (550 µL) were then transferred to 5 mm NMR tubes for analysis.

### 2.5. NMR Data Acquisition and Processing

NMR spectra were acquired on a Bruker Avance III HD 500 NMR spectrometer (University of Aveiro, Portuguese NMR Network, Aveiro, Portugal) operating at 500.13 MHz for ^1^H observation, at 298 K. One-dimensional (1D) 1H spectra were recorded with 32 k points, 7002.80 Hz spectral width, a 2 s relaxation delay, and 512 scans, using the pulse programs “noesypr1d” and “zg” for aqueous and lipidic samples, respectively. Spectral processing was carried out in TopSpin 4.0.3 (Bruker BioSpin, Rheinstetten, Germany), and consisted of cosine multiplication (ssb 2), zero-filling to 64 k data points, manual phasing, baseline correction, and calibration to TSP-d4/TMS signals (0 ppm). Two-dimensional (2D) NMR spectra, namely, 1H-1H TOCSY, J-resolved, and 1H-13C HSQC spectra, were also recorded for selected samples to aid metabolite identification. Signal assignment was based on matching 1D and 2D spectral information to reference spectra available in Chenomx 9.0 (Edmonton, AB, Canada), BBIOREFCODE-2–0–0 (Bruker Biospin, Rheinstetten, Germany), and HMDB.

### 2.6. Multivariate and Qualitative Analysis of Spectral Data

Multivariate analysis, namely principal component analysis (PCA) and partial least squares discriminant analysis (PLS-DA) was carried out in SIMCA-P 11.5 (Umetrics, Umeå, Sweden), using data matrices normalized by total area (after exclusion of suppressed water and residual solvent signals) and scaled to unit variance. The results were visualized through factorial coordinates (“scores”) and contributions (“loadings”), colored according to variable importance to the projection (VIP). The robustness of PLS-DA models was assessed through sevenfold internal cross-validation, based on the obtained predictive power (Q^2^). Loadings profiles were represented using the R software version 4.1.3 (R Core Team (2020). R: A language and environment for statistical computing. R Foundation for Statistical Computing, Vienna, Austria. URL http://www.R-project.org/).

To provide a quantitative measurement of metabolic variations, selected signals, representative of individual metabolites, were integrated with Amix-Viewer 3.9.15 (Bruker Biospin, Rheinstetten, Germany). Normalized signal areas were then used as input in Metaboanalyst 5.0 (http://www.metaboanalyst.ca/, accessed on 15 November 2022) or in GraphPad Prism (GraphPad Software, Inc., La Jolla, CA, USA) to represent the results graphically. Moreover, for each metabolite, the percentage of variation relative to controls was calculated along with the effect size (ES) [25] and statistical significance (*p*-value).

### 2.7. Quantification of Cytokine Production

Secretion of selected cytokines (IL-6, IL-8, TNF-α, IFN-γ, and VEGF) was assessed in medium samples using ELISA kits, according to the manufacturer’s instructions (Peprotech, London, UK). Furthermore, mRNA expression levels of IL-1β and IL-10 were assessed by RT-qPCR. In brief, RNA was extracted from macrophages using the NZY Total RNA Isolation kit (Nzytech, Lisbon, Portugal) following the manufacturer’s instructions. The synthesis of cDNA was performed using the NZY First-Strand cDNA synthesis kit, with 500 ng of total RNA per reaction. Real-time PCR reactions were prepared using NZYSpeedy qPCR green master mix and 400 nM of each primer pair (Supplementary Table S1). Three independent experiments were carried out (two for hypoxic conditions). The results were normalized using the HPRT1 housekeeping gene as reference and evaluated using the 2-ΔΔCt method.

### 2.8. Statistical Analysis

The statistical significance of differences between experimental groups was determined in GraphPad Prism (GraphPad Software, Inc., La Jolla, CA, USA). When comparisons were made between several groups, one-way analysis of variance (ANOVA), with Tukey’s multiple comparison tests, was employed. Otherwise, the unpaired two-tailed Student’s *t*-test for small sample sizes was used. Statistical differences were indicated as * *p* < 0.05, ** *p* < 0.01, *** *p* < 0.005, **** *p* < 0.001.

## 3. Results

### 3.1. Metabolic Composition of BC Cells-Conditioned Media (CM)

**Figure 2:**
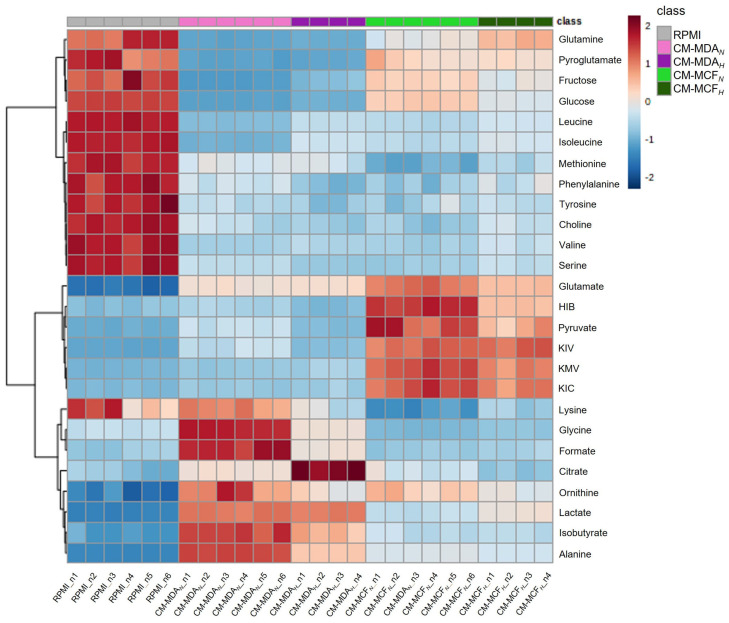
Heatmap summarizing the composition of the different media used in this work. RPMI: complete RPMI-1640 medium, used to incubate breast cancer cells and control macrophages; CM-MDA_N_: medium resulting from the normoxic culture of MDA-MB-231 cells; CM-MDA*_H_*: medium resulting from the hypoxic culture of MDA-MB-231 cells; CM-MCF*_N_*: medium resulting from the normoxic culture of MCF-7 cells; CM-MCF*_H_*: medium resulting from the hypoxic culture of MCF-7 cells. The different conditioned media (CM) were subsequently used for in vitro TEM generation. The color scale reflects the variations in metabolite levels, after normalization to total area and scaling to unit variance.

In order to obtain BC cells-conditioned media (CM), MDA-MB-231 and MCF-7 cells, which represent two distinct BC molecular subtypes, were cultured for 48 h either in normoxia or hypoxia. The two cell types profoundly and differentially changed the initial RPMI medium composition. Among the metabolites identified, 26 varied significantly between conditioned media (CM) and the RPMI control, as summarized in Figure 2 and in Supplementary Table S2. Both BC cell types consumed sugars, several amino acids, and choline, but to different extents. Glucose, fructose, glutamine, pyroglutamate, and the branched-chain amino acids (BCAA) leucine and isoleucine were more avidly consumed by MDA-MB-231 cells, whereas MCF-7 cells consumed higher amounts of methionine and lysine. Moreover, MCF-7 cells consumed glycine, which was secreted by MDA-MB-231 cells. Regarding the metabolites secreted to the culture medium, there were also clear differences between the two BC cell types. MDA-MB-231 cells secreted higher amounts of lactate, isobutyrate, citrate, formate, alanine, ornithine, and glycine (which was consumed by MCF-7), while MCF-7 cells secreted more pyruvate, glutamate, 2-hydroxybutyrate (HIB), and branched-chain ketoacids (BCKA), namely α-ketoisocaproate (KIC), α-ketoisovalerate (KIV), and α-keto-β-methylvalerate (KMV).

Cell incubation in a hypoxic environment exerted an additional modulation of the metabolites that were altered in the normoxic CM, in a cell type-dependent way. For instance, in MDA-MB-231 cells, hypoxia stimulated the consumption of serine, lysine, aromatic amino acids, and choline, whereas it diminished their use in MCF-7 cells. On the other hand, consumption of glucose and fructose was hardly affected by hypoxia in MDA-MB-231 cells but enhanced in hypoxic MCF-7 cells. In terms of the secretion pattern, hypoxia stimulated the release of citrate and KMV by MDA-MB-231 cells, as well as the secretion of lactate by MCF-7 cells. For the other secreted metabolites, hypoxia either had no impact or decreased their release into the culture medium.

In summary, the BC cells CM, collected for subsequent macrophage incubations, significantly differed in their composition. The CM-MDA*_N/H_* was richer in lactate, citrate (especially the hypoxic medium), formate, isobutyrate, and some amino acids (glycine, alanine, lysine), while the CM-MCF*_N/H_* contained higher amounts of glucose, fructose, pyruvate, BCKA, HIB, glutamine, glutamate, and pyroglutamate.

### 3.2. Exometabolomics of BC TEM

**Figure 3:**
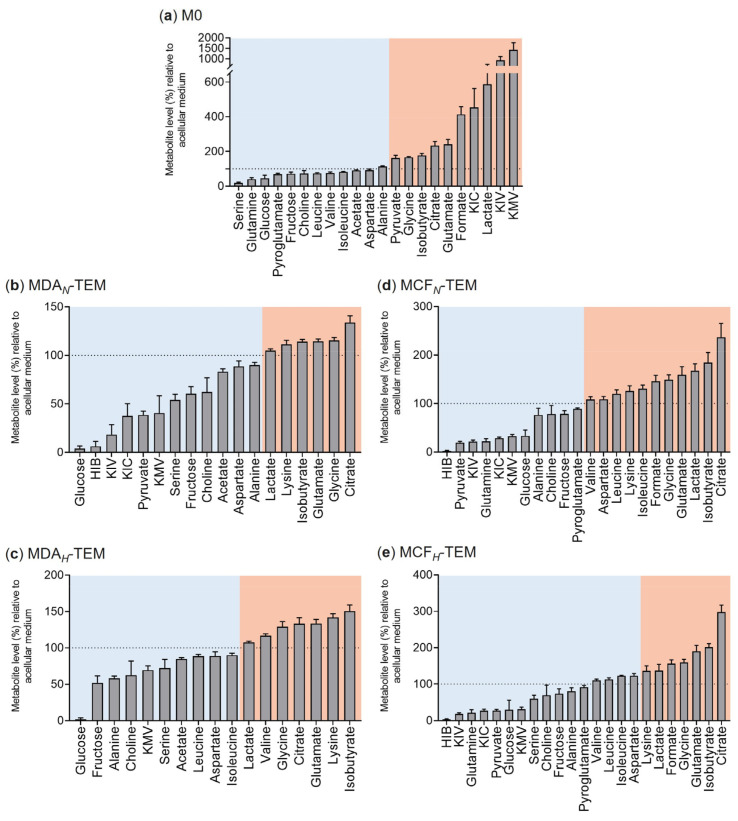
Extracellular metabolite levels in the culture medium of (**a**) control macrophages (M0) and (**b**–**e**) tumor-educated macrophages (TEM). The level of each metabolite is expressed as a percentage of the initial amount, present in the respective acellular medium (set to 100%, dashed line); hence, metabolites with levels below 100% were consumed by cells (blue shadow), whereas metabolites with levels above 100% were secreted (red shadow), upon 48 h in culture (Stage I).

Four subsets of tumor-educated macrophages (TEM) were generated in vitro through incubation of THP-1-derived macrophages, for 48 h, in CM from normoxic/hypoxic MDA-MB-231 or MCF-7 cells (MDA*_N_*-TEM, MDA*_H_*-TEM, MCF*_N_*-TEM, and MCF*_H_*-TEM). The consumption and secretion profiles of control macrophages (M0, cultured in standard RPMI medium) and of the different TEM subsets are shown in Figure 3 and in Supplementary Table S3.

The preferred substrates for M0 were serine, glutamine, and glucose, whose levels decreased, respectively, to 19%, 40%, and 46% of acellular medium, followed by pyroglutamate, fructose, choline, BCAA, acetate, and aspartate (Figure 3a). On the other hand, M0 released high amounts of BCKA, lactate, formate, glutamate, and citrate (more than twice the initial level), together with alanine, glycine, pyruvate, and isobutyrate (114–177% of initial level).

Compared to M0, MDA*_N_*-TEM showed several significant differences in their exometabolome (Figure 3b). Notably, several metabolites that had been secreted by tumor cells were used up by macrophages, namely 2-hydroxyisobutyrate (HIB), BCKA (KIV, KIC, KMV), pyruvate, and alanine. Additionally, MDA*_N_*-TEM consumed almost all the glucose present (<5% of the initial amount remaining), together with serine, fructose, and choline (54–62% of initial levels remaining). Small amounts of acetate and aspartate were also used up by MDA*_N_*-TEM. On the other hand, despite their availability in CM-MDA*_N_*, BCAA and pyroglutamate were not utilized by MDA*_N_*-TEM. Regarding the metabolites released by these macrophages, they included citrate, glutamate, isobutyrate, and glycine (secreted by MDA*_N_*-TEM in lower amounts than M0), together with lysine (not secreted by M0). Interestingly, the extracellular levels of lactate and formate remained practically unaltered.

Macrophages cultured in CM from hypoxic MDA-MB-231 cells (MDA*_H_*-TEM) displayed a metabolic activity broadly similar to that of MDA*_N_*-TEM, although some differences could be detected, namely in pyruvate, alanine, KIC, KIV, and HIB (Figure 3c). MDA*_H_*-TEM did not consume pyruvate (the levels of which were low CM-MDA*_H_*) but instead used up a greater amount of alanine. In addition, MDA*_H_*-TEM did not significantly consume KIV nor KIC and did not take up HIB, as this metabolite was absent from CM-MDA*_H_*.

The exometabolome profiles of macrophages incubated in CM from normoxic and hypoxic MCF-7 cells are shown in Figure 3d and Figure 3e, respectively. These profiles were similar to each other, with serine showing the only qualitative difference since it was available for consumption in CM-MCF*_H_*, but not in CM-MCF*_N_*. Both MCF*_N_*-TEM and MCF*_H_*-TEM consumed practically all the HIB present, together with BCKA and pyruvate, which decreased to 18–33% of the initial amounts present in the corresponding acellular media. These metabolites were available at high levels in CM from MCF-7 cells (Figure 2). Glucose and glutamine were also extensively consumed by MCF*_N/H_*-TEM, together with small amounts of fructose, alanine, and choline. It should also be noticed that, unlike M0 or MDA*_N/H_*-TEM, MCF*_N/H_*-TEM did not consume acetate nor aspartate, the latter having been secreted. Moreover, MCF*_N/H_*-TEM uniquely secreted BCAA, in line with the extensive use of BCKA by these cells. Other MCF*_N/H_*-TEM secretions comprised lysine (also observed in MDA*_N/H_*-TEM), lactate, and formate (weakly released by MDA*_N/H_*-TEM), together with citrate, isobutyrate, glutamate, and glycine (secreted by all macrophage subsets, although in different magnitudes).

**Figure 4:**
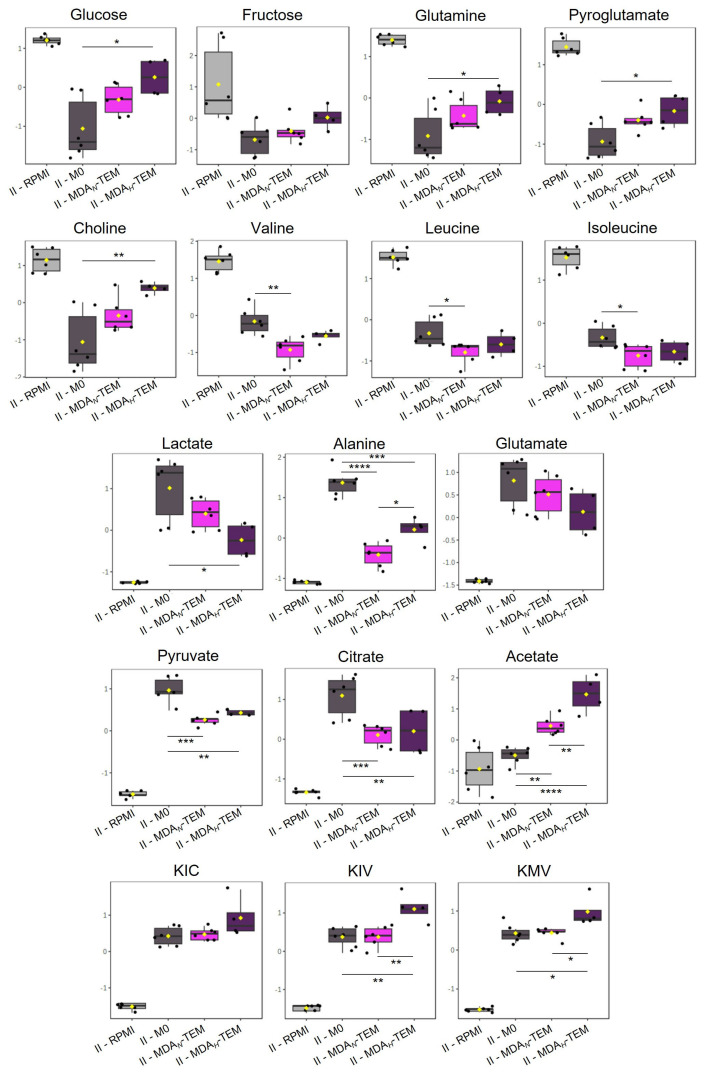
Relative levels of extracellular metabolites (scaled to unit variance) in acellular medium (RPMI), Stage II control macrophages (M0), and Stage II MDA*_N/H_*-TEM. Statistically significant differences between macrophages-conditioned media, as assessed by ANOVA, are indicated (* *p* < 0.05; ** *p* < 0.01; *** *p* < 0.005; **** *p* < 0.001).

Overall, the results presented above show that BC cells-CM had a substantial impact on macrophage metabolic activity, enforcing changes in their consumption and secretion patterns. To further assess whether cell metabolism was persistently reprogrammed, macrophages were incubated for an additional 48 h in a fresh RPMI medium (Stage II, Figure 1). The metabolomes of MDA*_N/H_*-TEM and MCF*_N/H_*-TEM, which had been cultured in BC cells-CM during Stage I, were then compared to the metabolome of M0 (cultured in RPMI in both Stages I and II). The exometabolomics results obtained for Stage II MDA*_N/H_*-TEM are shown in Figure 4 and in Supplementary Table S4, while the Stage II results for MCF*_N/H_*-TEM are presented in Figure S1 and in Supplementary Table S5. Stage II MDA*_N/H_*-TEM displayed significantly lower consumptions of glucose, glutamine, pyroglutamate, and choline than M0, together with the diminished secretion of lactate, alanine, pyruvate, glutamate (albeit not significant), and citrate. On the other hand, compared to Stage II M0, Stage II MDA*_N/H_*-TEM intensified the consumption of BCAA and secreted BCKA, although this latter change was only apparent for MDA*_H_*-TEM. Moreover, acetate (unchanged in Stage II M0) was released by MDA*_N/H_*-TEM. Regarding Stage II MCF*_N/H_*-TEM, they were characterized by significantly downregulated use of glucose, fructose, pyroglutamate, and choline, lower lactate secretion, and a trend for lower glutamine consumption and glutamate secretion. On the other hand, compared to Stage II M0, Stage II MCF*_N/H_*-TEM showed a trend to consume more isoleucine and secreted higher amounts of BCKA.

Hence, these results confirmed that culturing macrophages in BC cells-CM persistently affected their metabolism. In particular, their metabolic activity appeared to be reprogrammed towards downregulated glycolytic (glucose consumption, lactate secretion), and glutaminolytic (glutamine and pyroglutamate consumption, glutamate secretion) activities, along with altered BCAA/BCKA metabolism. Moreover, MDA*_N/H_*-TEM displayed specific differences in the secretion of pyruvate and citrate, likely related to TCA cycle modulation.

### 3.3. Endometabolomics of BC TEM

**Figure 5:**
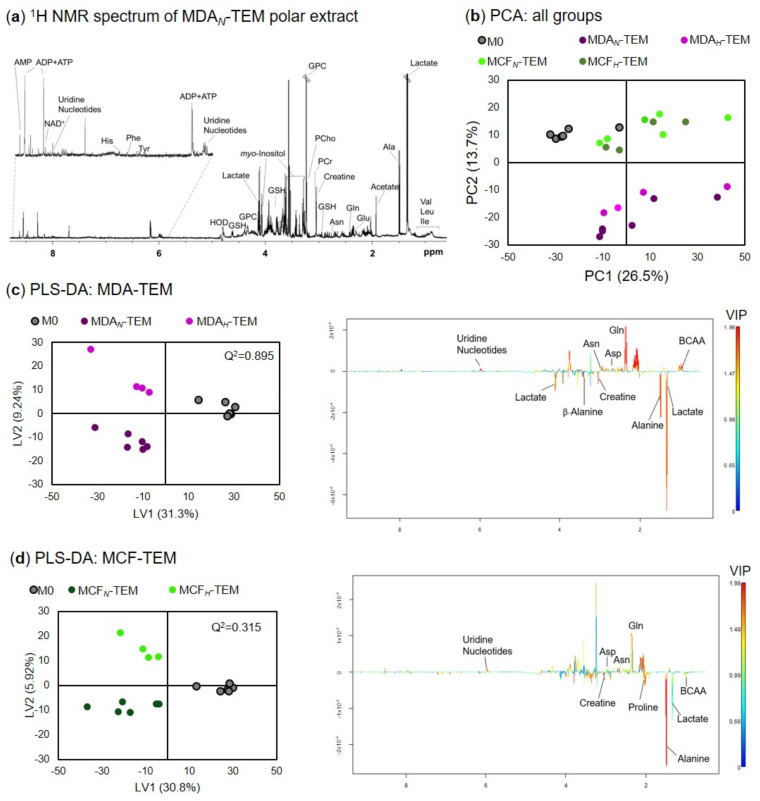
Macrophage endometabolomics: (**a**) representative ^1^H NMR spectrum of a polar extract from THP-1-derived macrophages, with some assignments, indicated (three letter codes used for amino acids, AMP/ADP/ATP—adenosine mono/di/triphosphate, GPC—glycerophosphocholine, GSH—glutathione, NAD^+^—nicotinamide adenine dinucleotide, PCho—phosphocholine, PCr—phosphocreatine); (**b**) PC1 vs. PC2 scores scatter plot obtained by principal component analysis (PCA) of the five sample groups compared; LV1 vs. LV2 scores (left) and LV1 loadings (right) plots obtained by partial least squares discriminant analysis (PLS-DA) of (**c**) MDA-TEM, and (**d**) MCF-TEM. The loadings profiles are colored according to variable importance to the projection (VIP).

^1^H NMR analysis of macrophage polar extracts enabled about 40 intracellular metabolites to be detected (Figure 5a), corroborating our previous report on the metabolome of THP-1-derived cells [26]. Principal component analysis (PCA) of spectral profiles showed a clear separation between M0 and TEM along PC1 (26.5% variance explained) (Figure 5b). Moreover, MDA*_N/H_*-TEM and MCF*_N/H_*-TEM separated in PC2 (13.7% variance), whereas a trend for distinguishing TEM cultured in normoxic vs. hypoxic CM was observed in PC3 (not shown). This distinction was further evident when each TEM subset was considered separately, the corresponding PLS-DA scores and loadings being shown in Figure 5c,d, for MDA-TEM and MCF-TEM, respectively.

**Figure 6:**
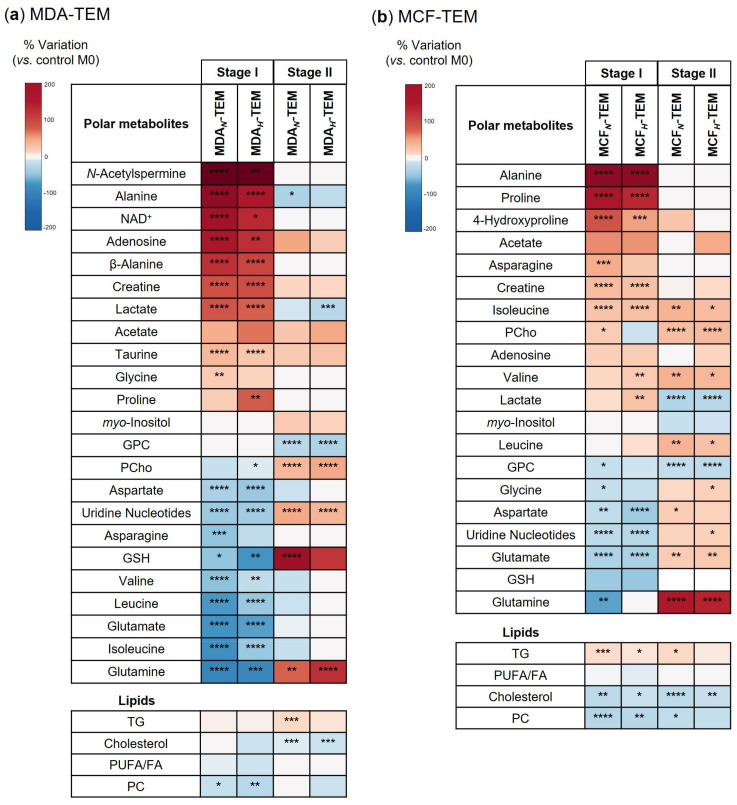
Heatmap summarizing the intracellular metabolite levels in (**a**) MDA-TEM and (**b**) MCF-TEM, expressed as % of variation relative to control M0. In each case, the first two columns correspond to macrophages incubated for 48 h in CM from BC cells cultured in normoxia/hypoxia (Stage I), while the 3rd and 4th columns correspond to cells re-incubated in fresh RPMI medium for an additional 48 h (Stage II). Statistical significance assessed in respect to controls (* *p* < 0.05; ** *p* < 0.01; *** *p* < 0.005; **** *p* < 0.001).

The main metabolites responsible for M0 vs. TEM discrimination are revealed in the LV1 loadings, colored according to variable importance to the projection (VIP). Notably, there are several differences between these signatures, not only in terms of magnitude but also regarding the direction of some variations. For instance, compared to M0, the levels of BCAA are suggested to be low in MDA-TEM (positive loadings in Figure 5c), and high in MCF-TEM (negative loadings in Figure 5d). This and other features were then verified through univariate statistics applied to quantitative variations of individual metabolites. The results are summarized in Supplementary Table S6 and in Figure 6, where the color scale is coded according to the % variation in each TEM type relative to M0 controls.

Compared to M0, Stage I MDA_N/H_-TEM contained higher levels of 11 polar metabolites, with N-acetylspermine, alanine, NAD^+^, adenosine, β-alanine, creatine, and lactate, being the most pronouncedly elevated (Figure 6a). On the other hand, 10 polar metabolites showed decreased intracellular levels in MDA_N/H_-TEM, the most marked reductions being seen for BCAA, glutamine, glutamate, and glutathione (GSH). Interestingly, some of these variations were reversed or assumed opposite directions when the cells were incubated for an additional 48 h in fresh RPMPI (Stage II). This was particularly noticeable for alanine, lactate, glutamine, GSH, phosphocholine (PCho), and uridine nucleotides (Figure 6a, 3rd and 4th columns in heatmap).

Furthermore, NMR analysis of the cell’s non-polar extracts provided complementary information on changes in some lipid constituents. Triglycerides (TG) showed a trend to increase, whereas cholesterol displayed a decreasing trend (Figure 6a). Phosphatidylcholine (PC) and polyunsaturated fatty acids (PUFA) were significantly lower in Stage I MDA*_N/H_*-TEM compared to M0. The changes in TG and cholesterol were even more pronounced at Stage II.

Regarding Stage I MCF*_N/H_*-TEM, the most upregulated intracellular metabolites were alanine, proline, and 4-hydroxyproline, whereas the highest decreases, in relation to M0 levels, were observed for glutamine, glutamate, and GSH (Figure 6b). Again, in stage II of the experiment (48 h in fresh medium, after 48 h incubation in CM), some differences between MCF*_N/H_*-TEM and M0 were attenuated, while others shifted direction (lactate, glutamine, glycine, aspartate, and uridine nucleotides). Finally, the changes in lipid molecules comprised increases in TG and decrease in cholesterol, PC, and PUFA, seen in both Stage I and Stage II (except for PUFA), as in the case of MDA-TEM.

### 3.4. Cytokine Production by CM-Generated TEM

**Figure 7:**
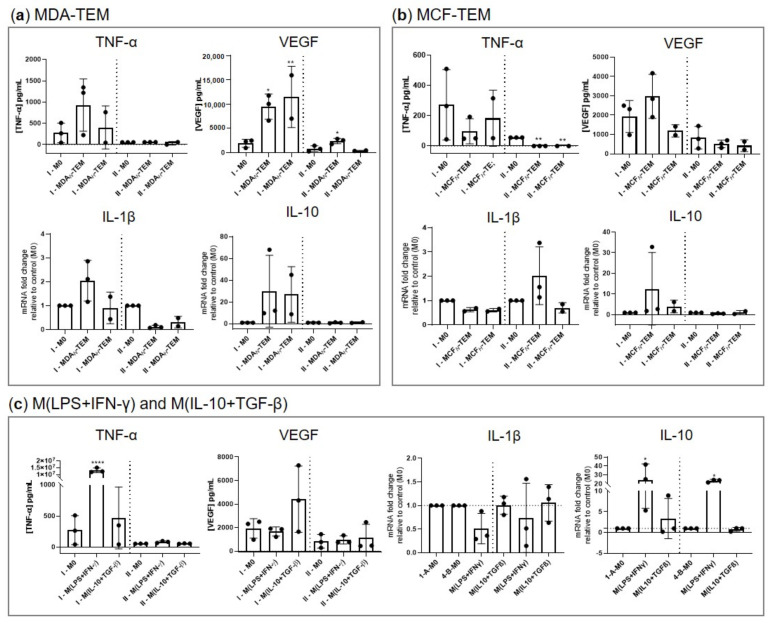
Cytokine production by (**a**) MDA-TEM, (**b**) MCF-TEM, (**c**) M(LPS+IFN-γ) and M(IL-10+TGF-β), in stages I and II (separated by the dashed line). Statistical significance assessed in respect to controls (* *p* < 0.05; ** *p* < 0.01; **** *p* < 0.001).

The functional impact of incubating macrophages with the different CM was analyzed by quantifying mRNA production and protein secretion for different cytokines, as shown in Figure 7. As a control, THP-1-derived macrophages were also incubated with either LPS and IFN-γ, cytokines that induce an M1-like phenotype, or with IL-10 and TGF-β, cytokines that can be found in the TME.

Importantly, MDA-TEM displayed significantly enhanced secretion of vascular endothelial growth factor (VEGF), a pro-angiogenic factor. This was maintained by MDA*_N_*-TEM in Stage II of the experiment, although to a smaller extent. On the other hand, mRNA expression of IL-1β, a pro-inflammatory cytokine, showed a trend for downregulation in Stage II, albeit not significant. As for MCF-TEM, the most striking difference consisted in the suppressed secretion of tumor necrosis factor α (TNF-α), a pro-inflammatory mediator, upon incubation in fresh RPMI medium for an additional 48 h (Stage II). The levels of mRNA for IL-10 did not show any significant differences in TEM, only showing significant increases in macrophages stimulated with LPS and IFN-γ. Finally, it should be mentioned that neither M0 nor TEM secreted detectable amounts of interleukin 6 (IL-6) and interferon-gamma (IFN-γ), two cytokines markedly produced upon macrophage pro-inflammatory stimulation (not shown). Taken together, these data suggest that MDA-TEM secreted VEGF while not producing significant amounts of pro-inflammatory mediators, while MCF-TEM showed a reduction in their capacity to produce TNF-α. In both cases, the changes pointed to a suppressive TME.

## 4. Discussion

**Figure 8:**
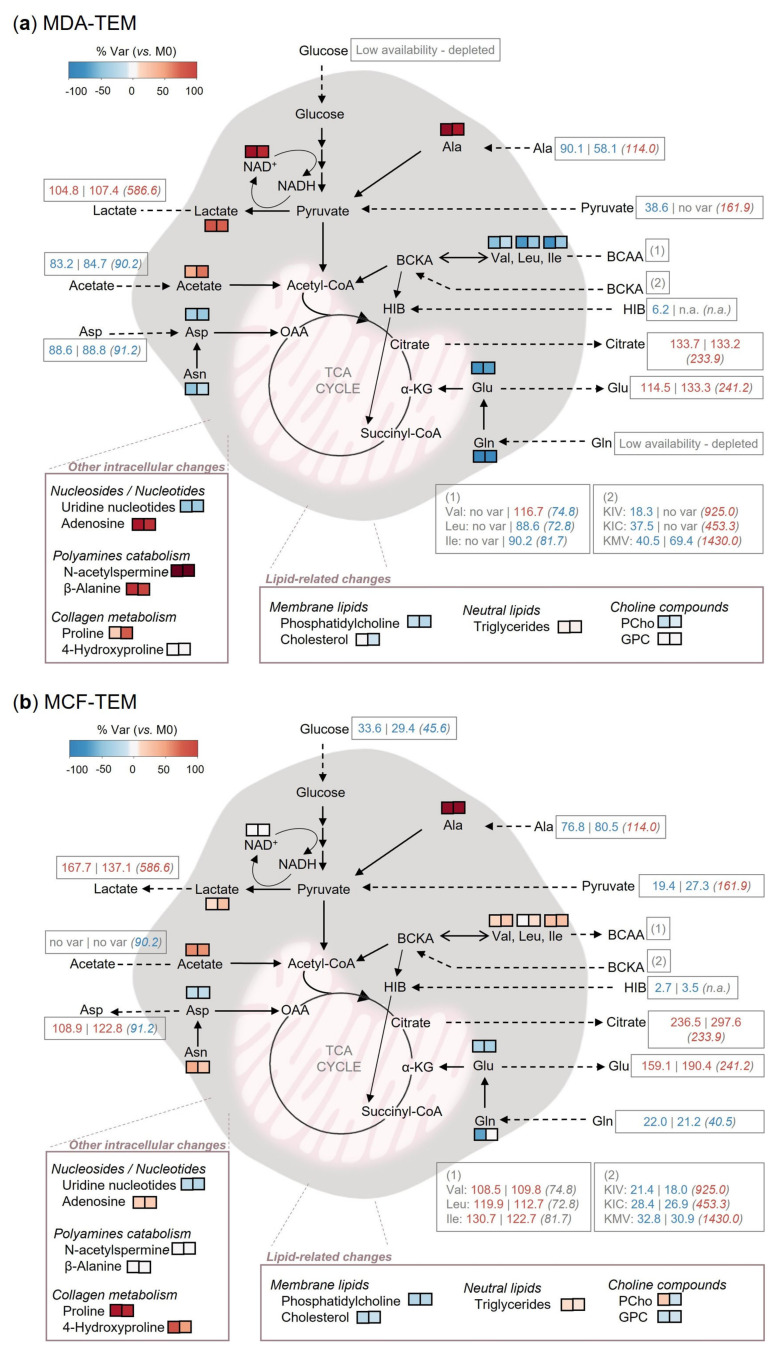
Summary of metabolic reprogramming observed for (**a**) MDA-TEM and (**b**) MCF-TEM. Intracellular changes are color-coded by the % variation in relation to M0, for TEM incubated in normoxic CM (left square) or hypoxic CM (right square), according to the heatmaps shown in Figure 6. To express extracellular variations, the percentages relative to the initial amounts in the respective acellular media (set to 100%) are shown for TEM incubated in normoxic CM (left) or hypoxic CM (right), as well as for M0 (in parenthesis). These variations are also shown in Supplementary Table S3, along with respective standard deviations.

Incubation of macrophages in the medium derived from tumor cell cultures has often been employed as a method to generate tumor-educated macrophages (TEM) in vitro [27,28,29,30,31,32]. However, despite the metabolic crosstalk between different cell types being recognized as a major event in the TME [14,15], little is known about the impact of tumor cells-CM on macrophage metabolism. In this work, we have assessed how THP-1-derived macrophages reprogram their metabolism upon incubation with CM from two BC cell lines (MDA-MB-231 and MCF-7), representative of different BC subtypes, cultured in normoxia or in hypoxia. Integration of exo- and endometabolomics profiling showed that the different CM finely modulated the metabolic activity of the in vitro generated TEM, as summarized in Figure 8 and discussed below.

The CM collected from BC cell cultures were characterized by varying contents of glucose and lactate, reflecting the different glycolytic activity of the two BC cell lines and its modulation by hypoxia. Glucose depletion and lactate enrichment were especially prominent in CM-MDA, indicating that MDA-MB-231 cells were more glycolytic than MCF-7 cells, in agreement with previous reports [33,34,35,36]. Tumor-derived lactate is known to critically influence both innate and adaptive immunity in the TME [37]. In macrophages, extracellular lactate has been reported to induce VEGF production, mediated by HIF-1α stabilization [16], as well as by hypoxia-independent mechanisms involving suppressed HIF-2α degradation due to mTORC1 activation [38]. The increased production of VEGF by MDA*_N/H_*-TEM observed herein is in line with this knowledge. In addition to high lactate, our in vitro generated TEM, especially MDA*_N_*_/*H*_-TEM, encountered a significant glucose shortage in the BC cells CM, which made them turn to the utilization of alternative fuels. These included pyruvate and alanine, released in high amounts by the BC cells. On the other hand, we found no evidence of lactate consumption, contrary to recent findings where extracellular lactate was postulated to support oxidative metabolism in isolated mice TAM [39] and in IL-4-stimulated M2 macrophages [40]. Indeed, extracellular lactate levels remained practically unchanged in the MDA*_N_*_/*H*_-TEM culture, while MCF*_N_*_/*H*_-TEM secreted some lactate, although to a much lower extent than control M0. Intracellularly, the increases in lactate and NAD^+^, especially prominent in MDA*_N_*_/*H*_-TEM, suggest active pyruvate to lactate conversion (catalyzed by lactate dehydrogenase, LDH), which can potentially imply lower pyruvate influx into the TCA cycle.

Interestingly, in Stage II, when extracellular glucose levels were restored by incubation in fresh RPMI medium, both MDA-TEM and MCF-TEM displayed lower glucose consumption and reduced intracellular and extracellular lactate, which suggests downregulation of glucose intake and/or glycolytic activity. These results are in contrast with other works where tumor extracts or cancer cells CM stimulated glycolysis in macrophages [27,41,42], suggesting glycolytic reprogramming strongly depends on the TAM-like model considered. Importantly, in vivo, a previous study assessing enzyme activities in situ has identified reduced glycolysis as a metabolic feature of human macrophages present in the microenvironment of cancerous colon tissue [43].

Exometabolomics further revealed that MDA-MB-231 cells, especially when cultured in hypoxia, and all macrophage subsets (M0 and TEM included) excrete citrate. Citrate secretion is a well-known feature of prostate epithelial cells [44] and astrocytes [45], where it is associated with specific physiological functions, but has seldom been reported for other cell types. Our observation that MDA-MB-231 cells, but not MCF-7 cells, secrete citrate may relate to the very recent findings by Grashei et al. showing increased pyruvate influx into the TCA cycle in MDA-MB-231 cells compared to MCF-7 cells [45]. Further, in that study, pyruvate-derived citrate was shown to partially originate from pyruvate carboxylation to oxaloacetate and reverse TCA cycle, which is in line with the high pyruvate carboxylase (PC) activity in BC tissues and MDA-MB-231 cells [46,47]. The role of PC in providing sufficient anaplerotic carbons to sustain citrate synthesis and secretion is further supported by a recent study in LNCaP prostate cancer cells [48]. Regarding the effect of hypoxia on citrate metabolism, it is interesting to note that previous work on TNBC cells has shown hypoxia to promote glycolytic fluxing into pyruvate and citrate (to the detriment of lactate) [49], which aligns with our observation that, compared to their normoxic counterparts, hypoxic MDA-MB-231 cells secreted more citrate (but not more lactate). Extracellular citrate is increasingly recognized to promote cancer cells’ growth, metastatic potential, and therapy resistance [50], raising the issue as to whether stromal cells in the TME can act as citrate providers. In support of this hypothesis, prostate cancer-associated fibroblasts (CAFs, generated through incubation in PC-3 cells CM) were shown to secrete citrate via the plasma membrane citrate transporter (pmCiC) [51]. Our results strongly suggest that human THP-1-derived macrophages are also equipped with this capability, calling for future in-depth studies on the extent and relevance of citrate secretion by TEM in the context of breast cancer.

The metabolism of BCAA, which is emerging as a critical player in cancer metabolism and immunity [49,52], showed striking differences between the cell subsets examined. Both MDA-MB-231 and MCF-7 cells consumed valine, leucine, and isoleucine while secreting their transamination products, the BCKA KIV, KIC, and KMV. BCAA to BCKA conversion is catalyzed by the branched-chain amino acid transaminases BCAT1 and BCAT2, the former being overexpressed in BC tissues [53] and in MDA-MB-231 cells, compared to normal breast epithelial cells [54]. BCKA can be further catabolized through an essentially irreversible reaction catalyzed by branched-chain ketoacid dehydrogenase (BCKDH), ultimately forming acetyl-CoA and succinyl-CoA, which can enter the TCA cycle for energy production [55] and/or lipid synthesis [56], as well as affect epigenetic regulation [52]. On the other hand, incomplete BCKA degradation can lead to their extracellular release, as recently observed for different cancer cell types [57,58,59]. The present study newly shows that MCF-7 cells excrete significantly more BCKA (and HIB, an intermediary in the KIV degradation pathway) than MDA-MB-231 cells. This is in contrast with the data reported by Kader et al., whereby highly metastatic BC cells released higher amounts of BCKA than BC cells with low metastatic potential [59]. However, MCF-7 cells were not included in that study, preventing direct comparison. In standard RPMI medium, THP-1-derived macrophages also consumed BCAA and secreted BCKA, although to a much lower extent than BC cells. However, when placed in CM from BC cells, macrophages switched to BCKA and HIB consumption, with MCF-TEM even secreting BCAA. The ability of human macrophages to take up BCKA and aminate them to BCAA has been previously demonstrated [57]. It was also shown that BCKA-exposed macrophages possessed decreased phagocytic capacity, potentially contributing to immune suppression in the TME [57]. Very recently, CM from lung cancer (LC) cells (enriched in BCKA), as well as exogenous BCKA, were further reported to influence the polarization of bone marrow-derived macrophages (BMDM) from mice [58]. Among other changes, they increased macrophage production of VEGF, similar to the results we obtained here for MDA*_N_*_/*H*_-TEM. In our study, however, CM from MCF-7 cells, which contained higher BCKA levels than CM from MDA-MB-231 cells, did not enhance VEGF production, suggesting the possible relationship between this pro-tumoral factor and BCKA exposure to be indirect or non-linear. On the other hand, Stage II MCF*_N_*_/*H*_-TEM decreased the secretion of the inflammatory factor TNF-α, also reported by Cai et al. upon incubation with CM from LC cells [58].

Glutamine, another important substrate for both cancer cells and macrophages, is involved in multiple metabolic pathways, including TCA cycle anaplerotic fueling, synthesis of glutathione (GSH, major intracellular antioxidant), and synthesis of nucleotides [60]. Compared to M0, all TEM subsets analyzed in this work displayed decreased intracellular levels of glutamine, glutamate, GSH, and uridine nucleotides, which may reflect the low glutamine availability in the BC cells CM. However, in Stage II, when extracellular glutamine levels were restored, these changes were reversed, with TEM showing higher levels of these metabolites. Interestingly, this cannot be justified through glutamine consumption, as it was lower in Stage II TEM vs. M0. One plausible hypothesis warranting future investigation regards the upregulation of glutamine synthetase (GS), which was identified in TAMs sorted from mice Lewis lung carcinoma (LLC) tumors [16], as well as in human TAMs isolated from glioblastoma tissues [61], and which was found to be intimately associated with pro-tumoral TAM polarization [62].

A distinctive feature in the intracellular profiles of MDA*_N_*_/*H*_-TEM regarded the elevated levels of *N*-acetylspermine and β-alanine. These metabolites may arise from the catabolism of polyamines, namely spermine, to prevent their excessive levels in cells [63]. Polyamines are produced from ornithine, which can be of extracellular origin (CM contained BC cells-secreted ornithine) and/or result from arginine conversion, catalyzed by Arginase 1 (Arg1). Upregulation of Arg1 is a known feature of anti-inflammatory M2-like macrophages [64], reported to be upregulated in isolated TAMs [16,65], as well as in TEM generated in vitro [32]. On the other hand, MCF*_N_*_/*H*_-TEM displayed high intracellular levels of proline and hydroxyproline, which are major constituents of collagen. Interestingly, a recent study has shown IL-4-stimulated macrophages to upregulate collagen uptake and degradation, resulting in the intracellular accumulation of collagen-derived free amino acids, which, in turn, upregulated arginine biosynthesis [66]. Our results suggest that similar events may occur in MCF*_N_*_/*H*_-TEM, the mechanisms and consequences of which require further investigation.

MDA*_N_*_/*H*_-TEM additionally showed high intracellular contents of adenosine, more prominent in Stage I, but still noted in Stage II. The immunosuppressive role of extracellular adenosine is well established, particularly via activation of A2_A_ and A2_B_ adenosine receptors in macrophages, which induces a pro-tumoral M2-like polarization [67]. However, little is known on the potential immunoregulatory role of intracellular adenosine. In a recent study, intracellular adenosine, accumulated through the inactivation of adenosine kinase (ADK, which converts adenosine to AMP), or translocated from the extracellular medium, was discovered to inhibit the inflammatory response in human endothelial cells (HUVECs) [68]. Remarkably, this effect was achieved by inhibiting the methylation of histone H3 at lysine 4 (H3K4), an epigenetic change known to regulate pro-inflammatory cytokine production in macrophages [69]. Hence, this is another example of a novel clue on the metabolic–phenotypic axis in TEM, provided by our metabolomics approach.

Regarding lipid-related variations, all TEM subsets displayed a trend for increased TG levels, which persisted in Stage II. This observation is in line with recent studies that detected high infiltration of lipid-loaded (“foamy”) TAMs in mice and human tumors, having identified the intracellular accumulation of neutral lipid-containing droplets (LDs) as a key determinant of the pro-tumoral activation and immune-suppressive functions of macrophages [70,71,72]. Moreover, MDA- and MCF-TEMs showed decreased cholesterol levels. Interestingly, membrane cholesterol efflux from macrophages was recently observed in TAMs isolated from ovarian cancer mice models and in macrophages incubated with CM from ID8 cancer cells [73]. That study robustly showed that cholesterol depletion from macrophages promoted IL-4-induced gene expression and tumor-supporting functions. Hence, it will be relevant to assess whether a similar phenomenon takes place in the context of the BC microenvironment. Finally, the variations in PC and PUFA, together with changes in the phospholipid-related metabolites PCho and GPC, suggest that BC cells’ CM stimulation of macrophages also interferes with their membrane composition and properties.

In sum, our results clearly revealed that macrophages are significantly and differentially affected by the metabolic conditions generated by the two BC subtypes studied, both at metabolic and phenotypic levels. To enhance the potential for clinical translation of these findings, follow-up studies should include more complex in vitro models, such as multicellular 3D culture systems that better mimic the pathophysiological situation. Indeed, the BC microenvironment comprises many cellular components [14], and, although BC cells and TAMs are dominant cells, other immune and non-immune stromal cells may influence the TME metabolic conditions. Ultimately, it will also be important to verify whether the phenotypic and metabolic plasticity of primary macrophages is similar to that of THP-1 cells. Notably, by identifying relevant metabolic pathways and metabolites in model cell lines, the present findings open the way to perform targeted and mechanistic studies in primary cells and in more complex cellular models.

## 5. Conclusions

Integration of exo- and endometabolomics profiling of in vitro generated TEM provided a detailed picture of the metabolic adaptations elicited in macrophages by incubation with BC cells-CM. These included the use of alternative substrates (alanine, pyruvate, BCKA), likely to compensate for glucose and glutamine shortage in the CM, and the reprogramming of several intracellular metabolic pathways, such as the catabolism of polyamines (in MDA-TEM), collagen degradation (more pronounced in MCF-TEM) and adenosine metabolism (mainly in MDA-TEM). Moreover, TEM changed their lipid composition in a way that possibly reflected lipid droplet buildup, cholesterol efflux, and membrane remodeling. Interestingly, following a second-stage incubation in fresh RPMI medium, TEM still displayed several metabolic differences compared to M0, which suggested downregulated glycolysis, increased glutamine and GSH synthesis (especially in MDA-TEM), and lipid metabolism reprogramming. Overall, this work provided new insights into the metabolic plasticity of TEM, revealing potentially important nutritional exchanges and immunoregulatory metabolites in the BC TME.

## Data Availability

The data supporting reported results are available from the corresponding author upon reasonable request.

## Supplementary Materials

The following supporting information can be downloaded at: https://www.mdpi.com/article/10.3390/cancers15041211/s1, Figure S1: Relative levels of extracellular metabolites (scaled to unit variance) in acellular medium (RPMI), Stage II control macrophages (M0) and Stage II MCF*_N_*_/*H*_-TEM; Table S1: Primers pairs used for RT-PCR: Table S2: Levels of extracellular metabolites in BC-cells conditioned media (CM), expressed as percentage of the initial amount in acellular complete RPMI medium (set to 100%); Table S3: Levels of extracellular metabolites in macrophage cultures incubated for 48 h (Stage I) in complete RPMI medium (M0), medium conditioned by normoxic MDA-MB-231 cells (MDA*_N_*-TEM), medium conditioned by hypoxic MDA-MB-231 cells (MDA*_H_*-TEM), medium conditioned by normoxic MCF-7 cells (MCF*_N_*-TEM), or medium conditioned by hypoxic MCF-7 cells (MCF*_H_*-TEM); Table S4: Levels of extracellular metabolites in cultures of control macrophages (M0) and of MDA*_N_*_/*H*_-TEM incubated for additional 48 h in fresh RPMI medium (Stage II); Table S5: Levels of extracellular metabolites in cultures of control macrophages (M0) and of MCF*_N_*_/*H*_-TEM incubated for additional 48 h in fresh RPMI medium (Stage II); Table S6: Intracellular variations in MDAN/H-TEM and MCFN/H-TEM, in Stages I and II, expressed in percentage (%) relative to respective controls (Stage I M0 and Stage II M0).
