# Supplementary material for: Metabolic Reprogramming of Breast Tumor-Educated Macrophages Revealed by NMR Metabolomics
Source: Cancers (Basel). 2023 Feb 14;15(4):1211. doi: 10.3390/cancers15041211 (PMC9954003; doi:10.3390/cancers15041211)
Supplement: Supplementary file 1 [file cancers-15-01211-s001.zip › cancers-2133184-supplementary.pdf]

# Supplementary Information

## Metabolic reprogramming of breast tumor educated macrophages revealed by NMR metabolomics

Ana S. Dias[1,2], Catarina R. Almeida[2], Luisa A. Helguero[2], Iola F. Duarte[1,*]

[1]CICECO - Aveiro Institute of Materials, Deparment of Chemistry, University of Aveiro, Aveiro, Portugal

[2]iBiMED - Institute of Biomedicine, Department of Medical Sciences, University of Aveiro, Aveiro, Portugal

*Correspondence: ioladuarte@ua.pt; Tel.: +351 234 401 418

**Figure S1.** Relative levels of extracellular metabolites (scaled to unit variance) in acellular medium (RPMI), Stage II control macrophages (M0) and Stage II MCF$_{N/H}$-TEM. Statistically significant differences between macrophages-conditioned media, as assessed by ANOVA, are indicated (* $p < 0.05$; ** $p < 0.01$).

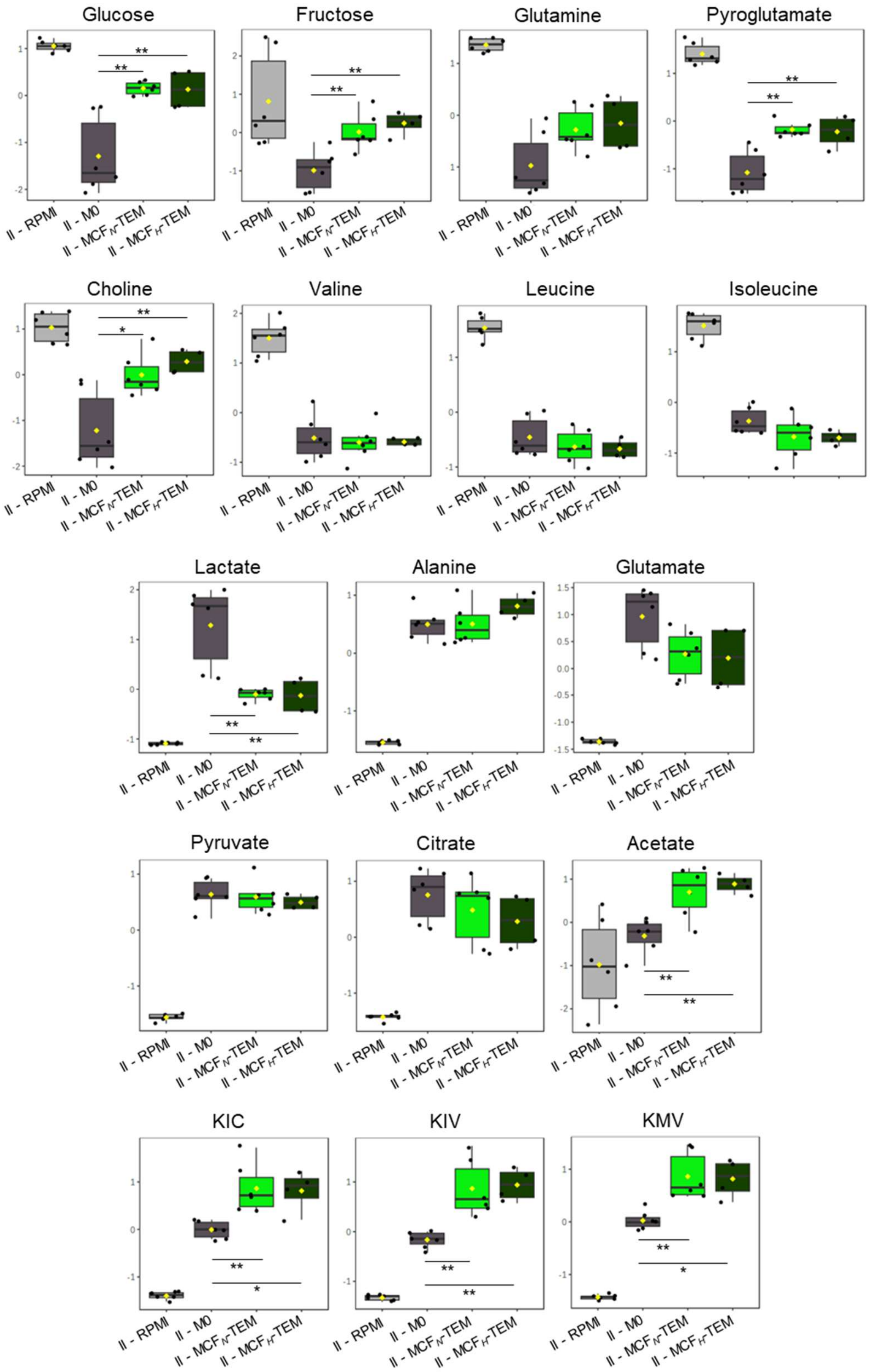

**Table S1.** Primers pairs used for RT-PCR.

| Genes | Forward Primer | Reverse Primer |
|---|---|---|
| HPRT1 | GCCATCTGCTTAGTAGAG | CTCAATAGTGCTGTGGTT |
| IL1b | GCTTGGTGATGTCTGGTC | GCTGTAGAGTGGGCTTATC |
| IL10 | ATAAGCTCCAAGAGAAAGGC | CCTGATGTCTCAGTTTCGTA |

**Table S2.** Levels of extracellular metabolites in BC-cells conditioned media (CM), expressed as percentage of the initial amount in acellular complete RPMI medium (set to 100%). CM-MDA$_N$: medium resulting from the normoxic culture of MDA-MB-231 cells; CM-MDA$_H$: medium resulting from the hypoxic culture of MDA-MB-231 cells; CM-MCF$_N$: medium resulting from the normoxic culture of MCF-7 cells; CM-MCF$_H$: medium resulting from the hypoxic culture of MCF-7 cells. One-way analysis of variance (ANOVA), with a Tukey's multiple comparison test, was employed for comparing the sample groups. The ANOVA results are presented in the four columns on the right (* $p < 0.05$; ** $p < 0.01$; *** $p < 0.005$; **** $p < 0.001$).

| | **% Relative to acellular complete RPMI (average ± SD)** | | | | **Significant differences** | | | |
|---|---|---|---|---|---|---|---|---|
| **Extracellular Metabolites** | **CM-MDA$_N$** | **CM-MDA$_H$** | **CM-MCF$_N$** | **CM-MCF$_H$** | **CM-MDA$_N$ *vs.* CM-MDA$_H$** | **CM-MCF$_N$ *vs.* CM-MCF$_H$** | **CM-MDA$_N$ *vs.* CM-MCF$_N$** | **CM-MDA$_H$ *vs.* CM-MCF$_H$** |
| **Alanine** | 672.5±7.3 | 468.5±1.4 | 350.2±4.7 | 331.3±0.6 | **** | *** | **** | **** |
| **Choline** | 50.4±3.9 | 44.1±2.3 | 43.7±3.2 | 52.4±2.4 | * | ** | * | ** |
| **Citrate** | 124.8±1.1 | 182.7±4.4 | 115.2±4.7 | 100.3±2.1 | **** | **** | *** | **** |
| **Formate** | 549.4±34.8 | 238.6±8.3 | 94.0±10.2 | 119.3±12.5 | **** | | **** | **** |
| **Fructose** | 48.3±0.4 | 54.9±1.2 | 77.9±1.2 | 69.7±2.3 | **** | **** | **** | **** |
| **Glucose** | 2.0±0.3 | 7.5±0.6 | 59.4±1.4 | 38.4±1.2 | **** | **** | **** | **** |
| **Glutamate** | 179.3±2.4 | 182.2±1.6 | 224.4±5.7 | 198.2±1.4 | | **** | **** | **** |
| **Glutamine** | 4.7±0.5 | 8.0±0.3 | 43.9±4.9 | 69.2±3.5 | | **** | **** | **** |
| **Glycine** | 302.3±6.2 | 143.9±1.4 | 49.0±2.3 | 62.5±1.5 | **** | *** | **** | **** |
| **HIB** | 305.4±46.1 | 42.6±37.7 | 2066.2±97.8 | 1161.5±26.0 | **** | **** | **** | **** |
| **Isobutyrate** | 382.7±15.3 | 291.3±19.7 | 182.7±11.3 | 181.7±5.8 | **** | | **** | **** |
| **Isoleucine** | 16.6±0.6 | 37.1±1.0 | 32.0±1.4 | 39.6±1.5 | **** | **** | **** | * |
| **KIC** | 266.7±48.4 | 320.0±80.0 | 2273.3±218.2 | 1930.0±170.9 | | * | **** | **** |
| **KIV** | 2325.0±352.2 | 900.0±122.5 | 7350.0±474.3 | 7500.0±474.3 | *** | | **** | **** |
| **KMV** | 790.0±110.1 | 1215.0±256.3 | 7590.0±540.3 | 6270.0±453.0 | | *** | **** | **** |
| **Lactate** | 993.8±20.9 | 974.7±12.5 | 473.4±20.9 | 630.0±16.2 | | **** | **** | **** |
| **Leucine** | 14.7±0.4 | 29.9±0.4 | 26.7±1.1 | 33.6±0.4 | **** | **** | **** | **** |
| **Lysine** | 99.8±4.2 | 73.3±6.8 | 51.5±2.9 | 65.7±3.0 | **** | *** | **** | |
| **Methionine** | 52.1±2.3 | 50.2±3.9 | 30.2±2.7 | 44.0±3.5 | | **** | **** | * |
| **Ornithine** | 258.6±26.2 | 196.9±13.3 | 218.6±12.1 | 187.2±6.6 | *** | | ** | |
| **Phenylalanine** | 57.7±2.1 | 45.7±2.8 | 49.2±3.8 | 58.1±4.5 | *** | ** | ** | *** |
| **Pyroglutamate** | 52.1±0.7 | 53.1±0.5 | 79.3±4.5 | 76.8±1.2 | | | **** | **** |
| **Pyruvate** | 439.8±34.5 | 173.9±11.8 | 1357.5±169.6 | 934.5±147.7 | * | *** | **** | **** |
| **Serine** | 29.2±1.2 | 19.1±0.9 | 21.2±2.2 | 30.1±2.5 | **** | **** | **** | **** |
| **Tyrosine** | 64.8±1.7 | 59.9±2.8 | 63.0±4.2 | 65.4±1.3 | | | | |
| **Valine** | 33.5±0.7 | 36.1±1.8 | 33.4±1.8 | 41.1±2.2 | | **** | | ** |

**Table S3.** Levels of extracellular metabolites in macrophage cultures incubated for 48h (Stage I) in: complete RPMI medium (M0), medium conditioned by normoxic MDA-MB-231 cells (MDA$_{N}$-TEM), medium conditioned by hypoxic MDA-MB-231 cells (MDA$_{H}$-TEM), medium conditioned by normoxic MCF-7 cells (MCF$_{N}$-TEM), or medium conditioned by hypoxic MCF-7 cells (MCF$_{H}$-TEM). The results are expressed as percentage of the initial amount in respective acellular media (set to 100%). One-way analysis of variance (ANOVA), with a Tukey's multiple comparison test, was employed for comparing the sample groups. The ANOVA results are presented in the four columns on the right (* $p < 0.05$; ** $p < 0.01$; *** $p < 0.005$; **** $p < 0.001$).

| | **% Relative to respective acellular media (average ± SD)** | | | | | **Significant differences** | | | |
|---|---|---|---|---|---|---|---|---|---|
| **Extracellular metabolites** | **M0** | **MDA$_{N}$-TEM** | **MDA$_{H}$-TEM** | **MCF$_{N}$-TEM** | **MCF$_{H}$-TEM** | **MDA$_{N}$-TEM *vs.* MDA$_{H}$-TEM** | **MCF$_{N}$-TEM *vs.* MCF$_{H}$-TEM** | **MDA$_{N}$-TEM *vs.* MCF$_{N}$-TEM** | **MDA$_{H}$-TEM *vs.* MCF$_{H}$-TEM** |
| **Acetate** | 90.2±4.1 | 83.2±3.0 | 84.7±2.0 | 99.2±5.0 | 98.2±3.2 | | | **** | *** |
| **Alanine** | 114.0±3.2 | 90.1±2.8 | 58.1±3.1 | 76.8±13.4 | 80.5±9.3 | **** | | | ** |
| **Aspartate** | 91.2±6.5 | 88.6±5.8 | 88.8±6.0 | 108.9±5.7 | 122.8±6.2 | | * | **** | **** |
| **Choline** | 72.4±19.9 | 62.3±14.5 | 62.5±19.4 | 78.4±17.7 | 69.1±28.0 | | | | |
| **Citrate** | 233.9±23.8 | 133.7±7.0 | 133.2±8.4 | 236.5±28.5 | 297.6±19.2 | | *** | **** | **** |
| **Formate** | 413.3±44.2 | 113.8±9.8 | 112.1±11.9 | 146.2±11.9 | 156.1±10.4 | | | | |
| **Fructose** | 71.3±9.1 | 60.4±7.4 | 51.8±9.6 | 78.7±6.5 | 73.3±13.8 | | | * | * |
| **Glucose** | 45.6±17.0 | 3.8±2.7 | 1.8±2.1 | 33.6±11.8 | 29.4±25.8 | | | * | |
| **Glutamate** | 241.2±27.8 | 114.5±2.3 | 133.3±5.9 | 159.1±17.0 | 190.4±15.7 | | | ** | ** |
| **Glutamine** | 40.5±8.8 | n.a. | n.a. | 22.0±5.5 | 21.2±8.4 | | | **** | **** |
| **Glycine** | 166.4±4.2 | 115.3±3.0 | 128.9±7.2 | 149.3±10.0 | 159.4±8.8 | * | | **** | **** |
| **HIB** | n.a. | 6.2±5.2 | n.a. | 2.7±0.8 | 3.5±1.5 | **** | | | **** |
| **Isobutyrate** | 176.9±11.9 | 114.1±2.3 | 150.5±8.6 | 184.2±20.9 | 201.6±9.5 | ** | | **** | *** |
| **Isoleucine** | 81.7±3.2 | 99.3±7.0 | 90.2±2.6 | 130.7±7.4 | 122.7±1.7 | | | **** | **** |
| **KIC** | 453.3±109.3 | 37.5±12.5 | 75.0±22.8 | 28.4±2.6 | 26.9±4.5 | | | | |
| **KIV** | 925.0±175.4 | 18.3±10.3 | 83.3±23.6 | 21.4±3.4 | 18.0±2.8 | | | | |
| **KMV** | 1430.0±336.0 | 40.5±17.8 | 69.4±5.8 | 32.8±3.3 | 30.9±5.9 | | | | |
| **Lactate** | 586.6±154.3 | 104.8±1.8 | 107.4±1.7 | 167.7±14.4 | 137.1±17.0 | | | | |
| **Leucine** | 72.8±4.6 | 98.3±4.6 | 88.6±2.6 | 119.9±8.2 | 112.7±4.0 | | | **** | **** |
| **Lysine** | 95.5±16.8 | 111.4±4.2 | 141.9±5.0 | 125.6±10.9 | 136.1±13.9 | ** | | | |
| **Pyroglutamate** | 68.8±5.5 | 98.7±2.1 | 103.3±2.7 | 89.0±1.7 | 91.4±4.7 | | | ** | ** |
| **Pyruvate** | 161.9±16.0 | 38.6±3.7 | 103.8±2.5 | 19.4±2.8 | 27.3±3.3 | **** | | ** | **** |
| **Serine** | 19.3±4.4 | 53.8±6.0 | 72.1±12.1 | n.a. | 59.8±9.7 | * | **** | **** | |
| **Valine** | 74.8±6.5 | 104.0±3.6 | 116.7±2.7 | 108.5±5.7 | 109.8±3.9 | ** | | | |

*n.a. not available*

**Table S4.** Levels of extracellular metabolites in cultures of control macrophages (M0) and of $MDA_{N/H}$-TEM incubated for additional 48h in fresh RPMI medium (Stage II). The results are expressed as percentage of the initial amount in acellular medium (set to 100%). One-way analysis of variance (ANOVA), with a Tukey's multiple comparison test, was employed for comparing the TEM groups. The ANOVA results are presented in the three columns on the right (* p < 0.05; ** p < 0.01; *** p < 0.005; **** p < 0.001).

| | **% Relative to acellular complete RPMI (average ± SD)** | | | **Significant differences** | | |
|---|---|---|---|---|---|---|
| **Extracellular metabolites** | **M0** | **$MDA_N$-TEM** | **$MDA_H$-TEM** | **$MDA_N$-TEM *vs.* M0** | **$MDA_H$-TEM *vs.* M0** | **$MDA_N$-TEM *vs.* $MDA_H$-TEM** |
| Acetate | 104.1±2.4 | 112.9±2.7 | 122.3±5.6 | ** | **** | ** |
| Alanine | 132.4±4.4 | 109.0±3.9 | 117.1±4.2 | **** | *** | * |
| Choline | 60.1±14.9 | 72.9±8.5 | 86.3±2.9 | | ** | |
| Citrate | 221.7±26.2 | 172.3±13.1 | 177.0±29.4 | ** | * | |
| Fructose | 82.5±5.1 | 85.2±3.8 | 89.5±3.8 | | | |
| Glucose | 59.1±14.3 | 72.5±7.1 | 82.9±8.6 | | * | |
| Glutamate | 329.4±57.5 | 297.9±45.7 | 258.3±52.6 | | | |
| Glutamine | 38.0±16.5 | 51.1±10.3 | 60.4±9.2 | | * | |
| Isoleucine | 80.9±2.7 | 76.6±2.8 | 77.5±2.7 | * | | |
| KIC | 1125.0±134.2 | 1150.0±90.8 | 1387.5±283.9 | | | |
| KIV | 1325.0±175.4 | 1325.0±175.4 | 1800.0±244.9 | | ** | ** |
| KMV | 1554.5±175.1 | 1563.6±101.6 | 1963.6±292.2 | | * | * |
| Lactate | 389.4±99.1 | 310.6±46.6 | 230.1±52.9 | | * | |
| Leucine | 79.5±3.7 | 74.3±2.9 | 76.5±3.2 | * | | |
| Pyroglutamate | 70.4±5.4 | 77.1±3.6 | 80.0±5.1 | | * | |
| Pyruvate | 287.3±22.7 | 234.2±10.1 | 246.8±4.4 | *** | ** | |
| Valine | 85.0±3.4 | 77.9±3.2 | 81.3±1.5 | ** | | |

**Table S5.** Levels of extracellular metabolites in cultures of control macrophages (M0) and of $MCF_{N/H}$-TEM incubated for additional 48h in fresh RPMI medium (Stage II). The results are expressed as percentage of the initial amount in acellular medium (set to 100%). One-way analysis of variance (ANOVA), with a Tukey's multiple comparison test, was employed for comparing the TEM groups. The ANOVA results are presented in the three columns on the right (* p < 0.05; ** p < 0.01).

| | % Relative to acellular complete RPMI (average ± SD) | | | Significant differences | | |
|---|---|---|---|---|---|---|
| **Extracellular metabolites** | **M0** | **$MCF_N$-TEM** | **$MCF_H$-TEM** | **$MCF_N$-TEM *vs.* M0** | **$MCF_H$-TEM *vs.* M0** | **$MCF_N$-TEM *vs.* $MCF_H$-TEM** |
| Acetate | 104.1±2.4 | 110.3±3.6 | 111.4±1.3 | ** | ** | |
| Alanine | 132.4±4.4 | 132.5±5.6 | 137.3±3.1 | | | |
| Choline | 60.1±14.9 | 81.6±8.1 | 86.8±4.7 | * | ** | |
| Citrate | 221.7±26.2 | 206.6±33.7 | 195.3±27.1 | | | |
| Fructose | 82.5±5.1 | 92.3±4.7 | 94.5±3.0 | ** | ** | |
| Glucose | 59.1±14.3 | 84.3±2.5 | 83.9±7.3 | ** | ** | |
| Glutamate | 329.4±57.5 | 260.8±44.2 | 253.5±58.3 | | | |
| Glutamine | 38.0±16.5 | 56.4±11.0 | 59.8±13.9 | | | |
| Isoleucine | 80.9±2.7 | 77.7±4.4 | 77.5±1.5 | | | |
| KIC | 1125.0±134.2 | 1762.5±381.7 | 1725.0±324.0 | ** | * | |
| KIV | 1325.0±175.4 | 2400.0±607.5 | 2475.0±357.1 | ** | ** | |
| KMV | 1554.5±175.1 | 2390.9±458.2 | 2345.5±364.5 | ** | * | |
| Lactate | 389.4±99.1 | 219.7±14.1 | 216.8±43.9 | ** | ** | |
| Leucine | 79.5±3.7 | 77.7±3.2 | 77.3±1.8 | | | |
| Pyroglutamate | 70.4±5.4 | 81.1±2.0 | 80.6±4.1 | ** | ** | |
| Pyruvate | 287.3±22.7 | 283.5±24.3 | 275.3±11.4 | | | |
| Valine | 85.0±3.4 | 84.3±2.7 | 84.4±0.5 | | | |

**Table S6.** Intracellular variations in $MDA_{N/H}$-TEM and $MCF_{N/H}$-TEM, in Stages I and II, expressed in percentage (%) relative to respective controls (Stage I M0 and Stage II M0).

| | **Stage I** | | **Stage II** | | **Stage I** | | **Stage II** | |
|---|---|---|---|---|---|---|---|---|
| **Intracellular Metabolites** | **$MDA_{N}$-TEM** | **$MDA_{H}$-TEM** | **$MDA_{N}$-TEM** | **$MDA_{H}$-TEM** | **$MCF_{N}$-TEM** | **$MCF_{H}$-TEM** | **$MCF_{N}$-TEM** | **$MCF_{H}$-TEM** |
| **Acetate** | 42.7±22.4 | 67.5±32.1 | 24.7±20.6 | 45.6±26.3 | 59.0±22.4 | 56.1±30.5 | 0 | 45.5±24.2 |
| **Adenosine** | 170.2±18.4 | 124.5±22.4 | 49.3±15.6 | 19.6±17.0 | 18.4±8.1 | 20.2±18.4 | 0 | 13.0±11.1 |
| **Alanine** | 298.5±4.5 | 162.6±6.7 | -33.4±5.8 | -22.0±13.0 | 314.6±6.7 | 206.2±4.6 | 0 | 0 |
| **Asparagine** | -49.3±9.2 | -20.4±9.7 | 0 | 0 | 43.9±9.3 | 24.5±12.5 | 0 | 0 |
| **Aspartate** | -34.7±4.4 | -45.1±3.9 | -13.1±8.3 | 0 | -19.6±7.6 | -41.1±2.8 | 19.9±8.1 | 15.3±6.5 |
| **β-Alanine** | 124.0±8.3 | 101.2±12.8 | 15.2±5.9 | 0 | -17.1±12.9 | 0 | -17.4±9.6 | -14.0±13.7 |
| **Creatine** | 90.3±2.9 | 92.0±3.8 | 13.2±3.9 | 11.7±7.4 | 33.4±1.9 | 29.1±2.2 | 5.7±3.9 | 9.7±6.8 |
| **Glutamate** | -75.7±3.7 | -68.5±3.1 | 6.7±4.0 | 12.8±5.2 | -31.6±3.6 | -33.6±5.9 | 20.3±4.2 | 23.1±6.6 |
| **Glutamine** | -97.0±18.1 | -92.2±14.7 | 74.5±7.7 | 124.5±7.0 | -66.8±15.1 | 0 | 154.6±9.3 | 139.2±3.0 |
| **GPC** | 0 | 0 | -25.0±3.5 | -31.5±3.8 | -16.8±5.9 | -10.3±8.0 | -18.9±1.3 | -21.7±1.1 |
| **Glycine** | 21.4±4.1 | 15.6±6.6 | 0 | 0 | -17.6±7.2 | -16.3±6.7 | 10.8±3.9 | 16.6±1.8 |
| **GSH** | -49.3±19.5 | -74.5±26.0 | 238.1±19.8 | 118.4±36.9 | -42.3±22.9 | -45.6±28.8 | 0 | 0 |
| **Hydroxyproline** | -28.3±6.7 | 0 | 0 | 16.4±14.2 | 87.7±8.1 | 50.8±9.5 | 25.2±5.7 | 0 |
| **Isoleucine** | -79.9±7.7 | -45.3±5.6 | -15.6±4.4 | 11.4±6.4 | 26.9±5.1 | 29.5±4.1 | 39.1±8.1 | 31.5±11.2 |
| **Lactate** | 85.8±4.1 | 74.8±7.9 | -9.8±3.6 | -25.3±4.4 | 9.5±4.1 | 27.8±5.3 | -29.1±3.3 | -26.2±2.7 |
| **Leucine** | -72.9±7.9 | -45.4±6.5 | -13.9±4.7 | 0 | 0 | 9.2±4.9 | 38.8±7.3 | 28.9±13.2 |
| **m-Inositol** | 10.3±5.0 | 0 | 21.5±5.6 | 15.0±10.9 | 0 | 0 | -16.0±9.3 | -11.3±9.5 |
| **N-acetylspermine** | 1160.9±28.9 | 743.9±63.1 | 0 | -56.0±37.1 | 168.7±36.8 | 239.1±51.5 | -53.4±33.2 | 0 |
| **$NAD^{+}$** | 215.1±20.1 | 132.1±30.5 | *n.a* | *n.a.* | 50.9±20.3 | 0 | *n.a* | *n.a* |
| **Phosphocholine** | -15.4±5.1 | -8.5±5.0 | 36.3±2.7 | 46.0±4.2 | 20.6±8.0 | -13.0±4.4 | 31.3±2.5 | 34.9±2.3 |
| **Proline** | 19.3±7.4 | 78.7±8.4 | -23.9±8.6 | 0 | 165.7±10.0 | 133.9±15.8 | 0 | 0 |
| **Taurine** | 36.2±1.4 | 25.5±3.2 | 22.3±5.4 | 27.6±12.4 | -7.1±2.3 | 0 | 9.6±7.1 | 21.2±11.8 |
| **Uridine nucleotide** | -44.1±2.6 | -40.6±3.1 | 45.8±4.7 | 37.5±5.6 | -22.6±3.1 | -26.7±1.7 | 14.4±4.9 | 16.8±5.8 |
| **Valine** | -51.2±4.7 | -20.1±6.2 | -15.5±3.9 | 14.1±8.7 | 11.4±4.5 | 21.5±7.0 | 40.8±10.0 | 35.5±13.4 |

*n.a. not available*
